# Supplementary material for: Characterization of coral-associated microbial aggregates (CAMAs) within tissues of the coral Acropora hyacinthus
Source: Sci Rep. 2019 Oct 10;9:14662. doi: 10.1038/s41598-019-49651-7 (PMC6787259; doi:10.1038/s41598-019-49651-7)
Supplement: Supplementary file 1 — Supplementary Information [file 41598_2019_49651_MOESM1_ESM.pdf]

Supplementary information

Characterization of coral-associated microbial aggregates (CAMAs) within tissues of the coral *Acropora hyacinthus*

Naohisa Wada[1,2,7], Mizuki Ishimochi[2,3], Taeko Matsui[2], F. Joseph Pollock[4,7,8], Sen-Lin Tang[1], Tracy D. Ainsworth[5], Bette L. Willis[6,7], Nobuhiro Mano[2,†], David G. Bourne[6,7,9 †]

[1]Biodiversity Research Center, Academia Sinica, Nangang, Taipei 11529, Taiwan
[2]Department of Marine Science and Resources, College of Bioresource Science, Nihon University, Fujisawa, Kanagawa 252-0813, Japan
[3]Faculty of Science, University of the Ryukyus, Nishihara, Okinawa 903-0213, Japan
[4]The Nature Conservancy, Caribbean Division, Coral Gables, FL 33134
[5]School of Biological, Earth and Environmental Science, University of New South Wales, Sydney, NSW 2052, Australia
[6]College of Science and Engineering, James Cook University, Townsville, QLD 4811, Australia
[7]AIMS@JCU, Townsville, QLD 4811, Australia
[8]Department of Biology, The Pennsylvania State University, State College, PA 16802, USA
[9]Australian Institute of Marine Science, Townsville, QLD 4810, Australia

†Equal correspond authors
Dr. Nobuhiro Mano (mano.nobuhiro@nihon-u.ac.jp) Department of Marine Science and Resources, College of Bioresource Science, Nihon University, Fujisawa, Kanagawa, 252-0813 Japan. Phone +81 466 84 3357
Dr. David G. Bourne (david.bourne@jcu.edu.au) College of Science and Engineering, James Cook University, Townsville, QLD, 4811 Australia. Phone +61 7 47814790

Suppl. table S1 Fisher's exact test values for the presence/absence of CAMA detected by HE stain and FISH among samples collected from the five sites

	Compared element	p-value of Adj. Fisher exact test
CAMAs detected by HE[†]	Sesoko Is. vs. Inner Shelf	0.6770
	Sesoko Is. vs. Lizard Is.	0.0155**
	Sesoko Is. vs. Outer Shelf	0.0151**
	Sesoko Is. vs. Orpheus Is.	0.0360**
	Inner Shelf vs. Lizard Is	0.1400
	Inner Shelf vs. Outer Shelf	0.1340
	Inner Shelf vs. Orpheus Is.	0.2830
	Lizard Is. vs. Outer Shelf	1.0000
	Lizard Is. vs. Orpheus Is.	1.0000
	Outer Shelf vs. Orpheus Is.	0.7980
CAMAs detected by FISH	Sesoko Is. vs. Inner Shelf	1.00000
	Sesoko Is. vs. Lizard Is.	0.00517***
	Sesoko Is. vs. Outer Shelf	0.00378***
	Sesoko Is. vs. Orpheus Is.	0.00357***
	Inner Shelf vs. Lizard Is	0.00517***
	Inner Shelf vs. Outer Shelf	0.00378***
	Inner Shelf vs. Orpheus Is.	0.00357***
	Lizard Is. vs. Outer Shelf	1.00000
	Lizard Is. vs. Orpheus Is.	1.00000
	Outer Shelf vs. Orpheus Is.	1.00000

[†]The HE stained CAMAs include basophilic and eosinophilic CAMAs.
P values are $**p < 0.05$ and $***p < 0.01$.

Suppl. table S2 Summary of the densities of CAMAs in HE- and FISH- stained tissues

	Sesoko Is.	Inner Shelf	Lizard Is.	Outer Shelf	Orpheus Is.
Sample size	10	10	10	8	10
**Basophilic CAMAs**					
Colonies observed with CAMAs	10	8	3	0	3
Ave. densities (n/cm^2) in tissues	20.13±17.1	6.78±9.3	0.92±0.1	-	3.55±3.3
Density range (n/cm^2) across samples	1.06 - 48.90	0.86 - 25.48	0.87 - 1.02	-	1.54 - 7.42
**Eosinophilic CAMAs**					
Colonies observed with CAMAs	0	1	0	2	1
Ave. densities (n/cm^2) in tissues	-	0.86	-	5.39±6.7	1.28±2.8
Density range (n/cm^2) across samples	-	-	-	0.69 - 10.10	
**FISH detected CAMAs**					
Colonies observed with CAMAs	10	10	3	2	2
Ave. densities (n/cm^2) in tissues	18.72±12.7	7.90±8.2	1.26±0.6	0.90±0.5	4.48±4.2
Density ranges (n/cm^2) across samples	0.53 - 41.08	0.86 - 25.48	0.87 - 1.02	0.58 - 1.22	1.54 - 7.42

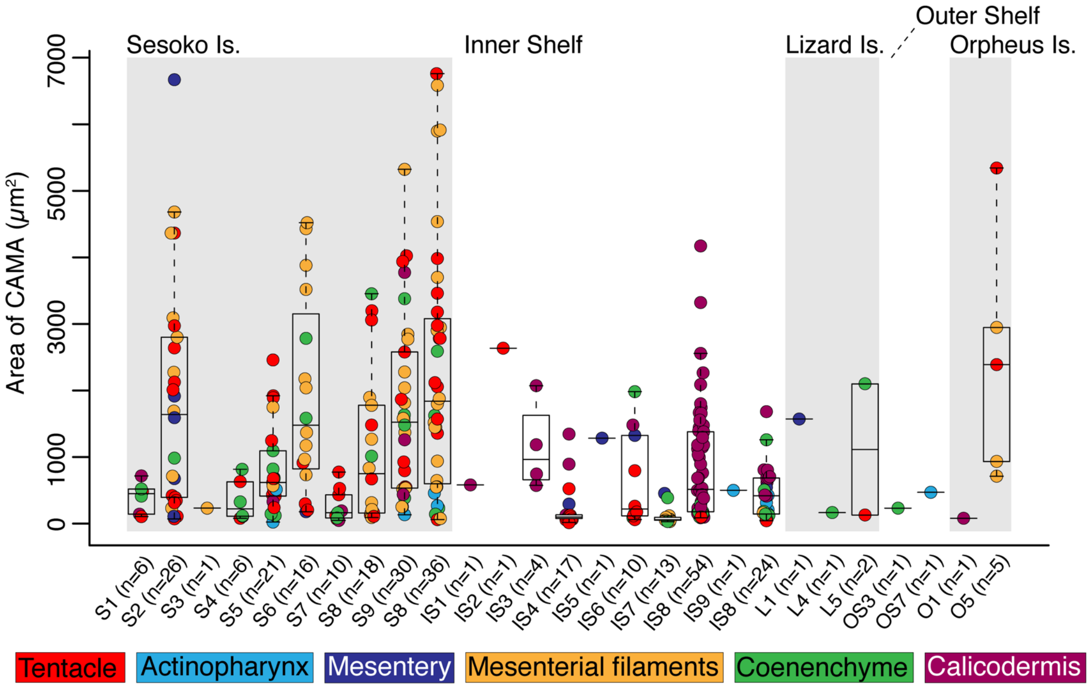

Suppl. Fig. S1 High variation of CAMA size at the individual colony level. Dot plots with box plots showing the size of CAMAs among six anatomical regions (see color coding in below index) for each site.

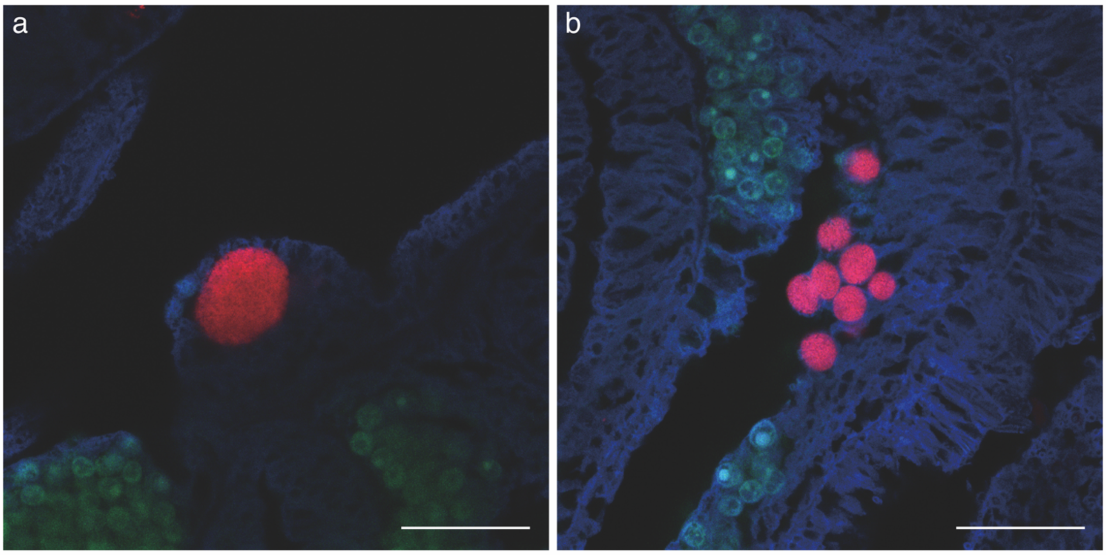

Suppl. Fig. S2 Calculating the area of CAMAs. (a) A single large CAMA, comprised of rod-shaped bacteria, was calculated to be 175.7 μm^2 in cross-sectional area from 3D images. **(b)** Smaller, numerous aggregations were calculated to be, on average, 22.6±4.2 μm^2 in cross-sectional area (n = 8). Scale bars indicate 50 μm.

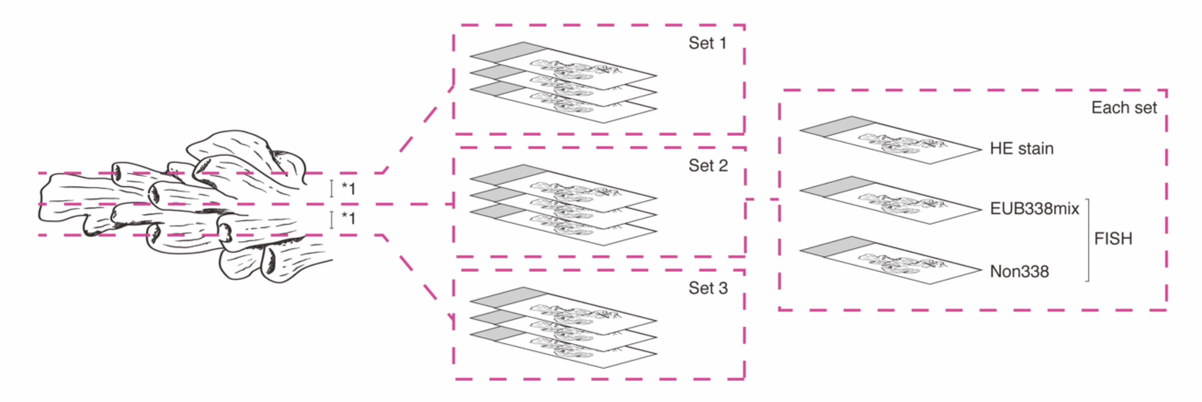

Suppl. Fig. S3 Schematic drawing showing how coral fragments were sectioned for HE staining and FISH. In total, nine sections were collected from each sample (three sets of sections, each set comprised of three serial sections). *1: Distance between each set was 100 μm.
